# Modification of Thin Film Composite PVA/PAN Membranes for Pervaporation Using Aluminosilicate Nanoparticles

**DOI:** 10.3390/ijms23137215

**Published:** 2022-06-29

**Authors:** Katsiaryna S. Burts, Tatiana V. Plisko, Vladimir G. Prozorovich, Galina B. Melnikova, Andrei I. Ivanets, Alexandr V. Bildyukevich

**Affiliations:** 1 Institute of Physical Organic Chemistry, National Academy of Sciences of Belarus, 220072 Minsk, Belarus; burt@ifoch.bas-net.by (K.S.B.); uf@ifoch.bas-net.by (A.V.B.); 2 Institute of General and Inorganic Chemistry, National Academy of Sciences of Belarus, 220072 Minsk, Belarus; vladimirprozorovich@gmail.com (V.G.P.); andreiivanets@yandex.by (A.I.I.); 3 A. V. Luikov Heat and Mass Transfer Institute, National Academy of Sciences of Belarus, 220072 Minsk, Belarus; galachkax@gmail.com

**Keywords:** thin film nanocomposite membrane, aluminosilicate nanoparticles, polyvinyl alcohol, dynamic membrane, pervaporation, ethanol dehydration

## Abstract

The effect of the modification of the polyvinyl alcohol (PVA) selective layer of thin film composite (TFC) membranes by aluminosilicate (Al_2_O_3_·SiO_2_) nanoparticles on the structure and pervaporation performance was studied. For the first time, PVA-Al_2_O_3_·SiO_2_/polyacrylonitrile (PAN) thin film nanocomposite (TFN) membranes for pervaporation separation of ethanol/water mixture were developed via the formation of the selective layer in dynamic mode. Selective layers of PVA/PAN and PVA-Al_2_O_3_·SiO_2_/PAN membranes were formed via filtration of PVA aqueous solutions or PVA-Al_2_O_3_·SiO_2_ aqueous dispersions through the ultrafiltration PAN membrane for 10 min at 0.3 MPa in dead-end mode. Average particle size and zeta potential of aluminosilicate nanoparticles in PVA aqueous solution were analyzed using the dynamic light scattering technique. Structure and surface properties of membranes were studied using scanning electron microscopy (SEM), atomic force microscopy (AFM) and water contact angle measurements. Membrane performance was investigated in pervaporation dehydration of ethanol/water mixtures in the broad concentration range. It was found that flux of TFN membranes decreased with addition of Al_2_O_3_·SiO_2_ nanoparticles into the selective layer due to the increase in selective layer thickness. However, ethanol/water separation factor of TFN membranes was found to be significantly higher compared to the reference TFC membrane in the whole range of studied ethanol/water feed mixtures with different concentrations, which is attributed to the increase in membrane hydrophilicity. It was found that developed PVA-Al_2_O_3_·SiO_2_/PAN TFN membranes were more stable in the dehydration of ethanol in the whole range of investigated concentrations as well as at different temperatures of the feed mixtures (25 °C, 35 °C, 50 °C) compared to the reference membrane which is due to the additional cross-linking of the selective layer by formation hydrogen and donor-acceptor bonds between aluminosilicate nanoparticles and PVA macromolecules.

## 1. Introduction

Pervaporation is an effective membrane process of liquid–liquid mixture separation due to a number of advantages: low energy consumption, ecological safety, no use of additional chemical reagents, possibility of separation of azeotropic mixtures, mixtures of isomers and substances with close boiling points [1,2]. Driving force in pervaporation is the difference in chemical potential between supramembrane and submembrane areas.

There is a growing interest in dehydration of organic substances, like ethanol, isopropanol and ethyl acetate, via pervaporation in industry [3]. Dehydrated ethanol is of the greatest interest among them due to the numerous areas of application: it is used in the production of cosmetics, detergents, coatings, polymers and chemicals, inks, pharmaceuticals, in medicine [3], as well as an additive to the automotive fuel to reduce carbon dioxide emissions [4]. Separation of ethanol/water mixture is challenging due to the formation of azeotrope at 95.6 wt.% of ethanol. Usually, dehydrated ethanol is produced by distillation, but it is arduous due to the balance of liquid and vapor phases in azeotrope. In this case pervaporation is a promising approach to separate such mixtures.

Polymeric membranes with dense selective layers are widely applicable for pervaporation. Hydrophilic membranes based on chitosan [5], sodium alginate [6], polyamide [7], polyvinyl alcohol (PVA) [8], are predominantly applied in hydrophilic pervaporation. However, beside the material’s nature, membrane selective layer characteristics are important (hydrophilic–hydrophobic balance, roughness, cross-linking degree). The trade-off between flux and selectivity is one of the significant problems in membrane material science [9]. One of parameters that can be optimized to obtain effective membranes is the membrane selective layer thickness. It is known that membrane flux in pervaporation is inversely proportional to selective layer thickness. Thus, membrane flux could be enhanced by decreasing selective layer thickness.

Thin film composite (TFC) membranes are widely used in membrane processes due to the thin selective layer that ensures higher flux together with high selectivity and higher mechanical strength which is provided by porous membrane support [10,11,12]. There are some frequently used methods of TFC membrane preparation: layer-by-layer technique [13]; solution casting [14]; dip, spin or spray coatings [15,16,17]; interfacial polymerization [18]; dynamic mode technique [19]. A selective layer of TFC membranes obtained in dynamic mode is formed via filtration of polymer or colloid solution through the porous membrane-substrate due to concentration polarization. Membrane material that is used for selective layer formation affects the selective layer thickness, separation performance as well as membrane mechanical properties [20]. A variety of membrane materials were reported for the formation of the selective layer of TFC dynamic membranes, such as activated carbon, metal-containing compounds, nanoparticles and polymers [21]. Composite dynamic membranes can be obtained via filtration of the solution or dispersion in cross-flow or dead-end modes [22,23]. The advantages of selective layer formation in the dynamic mode are (1) the need to use a small quantity of reagents; (2) short formation time of thin film layer; (3) simplicity of the formation process; (4) the possibility of real-time membrane characterization; (5) single-stage process; (6) the possibility of adapting the selective layer thickness and structure to the certain membrane process. One more advantage of preparation of TFC membranes via the dynamic mode is the possibility to form a selective layer on the surface of porous membranes which are assembled into modules. It will simplify the manufacturing technology of TFC membrane modules and prevent the damage of the selective layer during operations with membranes while modules are designed [24].

There are a number of advantages of dynamic mode [25,26] that were reported for preparation of membranes for microfiltration [27], ultrafiltration [28], nanofiltration [29], reverse osmosis [30], pervaporation [19]. Dependences of membrane structure and pervaporation performance in 90 wt.% ethanol/10 wt.% water feed mixture separation on polymer concentration, component ratio, filtration time, and transmembrane pressure upon TFC membrane formation via dynamic mode technique (dead-end ultrafiltration) were reported in our previous study [21].

However, decreasing the selective layer thickness is sometimes not enough to improve membrane performance. It is worth noting that concentration polarization can limit the component transport in pervaporation in the case of TFC membranes with very thin selective layers [31,32]. Modification of the selective layer is a promising technique to improve membrane transport properties. Rather often it is implemented by introduction of different nanofillers into the polymer membrane matrix to obtain nanocomposite membranes. Carbon nanotubes (as well functionalized) [33], graphene oxide [34], metal-organic frameworks (MOFs) [35] and covalent organic frameworks (COFs) [36], fullerene [8] and fullerene derivatives [37], etc., are used for organic polymer matrix modification.

It was reported that such additives like polyhedral oligomeric silsesquioxane nanocages, functional graphene oxide, silica, calcium oxalate, zeolitic imidazolate frameworks and 2D-nanomaterials yield the increase of total fluxes in pervaporation [38,39,40,41,42,43,44,45]. However, in some cases the increase in nanoparticle concentration leads to significant decrease in the selectivity of the nanocomposite membrane.

Aluminosilicates are one of the most widely used fillers that are introduced into the polymer materials to improve mechanical strength, heat and flame resistance, and barrier properties [46]. Aluminosilicate minerals belong to a family of materials with the chemical composition Al_2_SiO_5_ with Al–O–Si bonds and different crystal structures [47]. A big number of natural aluminosilicates have been extensively studied for various applications such as development of ceramics, glasses, adsorbents, fuel cells, artificial soil, synthetic zeolites, materials for building, catalysts [48,49,50]. Synthetic aluminosilicates have a number of advantages compared to natural aluminosilicates: higher purity and uniformity of structure, the possibility to adjust structure and properties according to application [47].

It was shown that mesoporous/microporous molecular sieves/clay aluminosilicates nanoparticles are promising additives to polymer membrane matrix to improve transport properties in ultrafiltration [51], nanofiltration [52], reverse osmosis [53,54], pervaporation [55,56,57,58], and gas separation. It was found that modification using aluminosilicate nanoparticles allowed for obtaining high-performance aluminosilicate-Nafion hybrid membranes with improved proton conductivity for direct methanol fuel cells [59,60]. Baroña et al. [54] presented a new approach of thin film nanocomposite (TFN) membrane preparation by introduction of hydrophilic single-walled aluminosilicate nanotubes (AS-SWNT) into the polyamide selective layer for low pressure reverse osmosis. Synthetic AS-SWNT (analogue of nanotubular mineral imogolite) was widely applied in different areas as a modifier. It is composed of a tubular aluminum (III) hydroxide layer on the outer surface with pendant silanol groups on the inner surface [61]. It was shown that developed nanocomposite membranes were characterized by enhanced permeability and rejection (water flux increased two times, rejection—from 95.6% to 96.2%). AS-SWNT were incorporated into the PVA selective layer to obtain thin film nanocomposite (TFN) membranes for nanofiltration with high flux and salt rejection [52]. The water flux of the developed TFN membranes was found to be 3–5 times higher compared to the reference unmodified membrane [52]. To study the effect of initial dispersion of AS-SWNT, Kang et al. [56] prepared nanocomposite PVA/AS-SWNT dense pervaporation membranes via two approaches: introduction of AS-SWNT powder or AS-SWNT gels to PVA aqueous solution at high loading (up to 40 vol.%). It was found that AS-SWNT gel addition to PVA aqueous solution yields substantially higher dispersion degree compared to powder addition. The significant increase in water flux through the membrane in pervaporation dehydration of ethanol and a moderate decrease of water/ethanol selectivity was revealed with the increase in AS-SWNT concentration in PVA membrane matrix. Dense PVA flat sheet membranes for pervaporation dehydration of 1,4-dioxane were modified by incorporation of sodium aluminosilicate aqueous dispersion into the PVA solution (2.5 wt.% and 7.5 wt.% sodium aluminosilicate in relation to PVA weight) and cross-linked using maleic acid [57]. It was found that both flux and separation factor increased with increasing sodium aluminosilicate content [57].

In this article for the first time TFN PVA-Al_2_O_3_·SiO_2_/PAN membranes were developed via the formation of a selective layer using dynamic mode technique (dead-end ultrafiltration mode). The effect of aluminosilicate nanoparticle concentration on selective layer structure and pervaporation performance of TFN dynamic membranes was studied. The effect of feed composition and temperature on the performance of developed PVA/PAN TFC and PVA-Al_2_O_3_·SiO_2_/PAN TFN membranes was investigated in pervaporation of pure water, pure ethanol and ethanol/water mixtures with water concentration 10, 20, 50, 80 wt.% and in pervaporation of 90 wt.% ethanol/10 wt.% water mixture at different feed temperatures: 25, 35 and 50 °C.

## 2. Results and Discussion

### 2.1. Average Size and Zeta Potential of Aluminosilicate Nanoparticles in PVA Aqueous Solution

The average size and zeta potential of aluminosilicate nanoparticles in PVA aqueous solution are presented in Table 1. The average size of Al_2_O_3_·SiO_2_ nanoparticles in 1 wt.% PVA aqueous solutions was found to significantly increase compared to the initial size in hydrosol (39 nm). This is probably the result of PVA shell formation around nanoparticles due to the hydrogen bonding between hydroxyl groups of PVA and silanol groups on the surface of nanoparticles, as well as donor–acceptor bonding between the undivided oxygen electron pairs of hydroxyl groups of PVA and free orbitals of aluminum atoms. It was observed that an increase in concentration of Al_2_O_3_·SiO_2_ nanoparticles led to monotonical increase in the average size of nanoparticles, which may be related to the formation of nanoparticle aggregates (Table 1).

It was found that the zeta potential of PVA dispersed nanoparticles in general became lower in absolute value compared to that of nanoparticles in the initial hydrosol (−7.1 mV), due to the nanoparticle surface charge screening by a shell of PVA macromolecules.

### 2.2. Scanning Electron Microscopy

**Figure 1:**
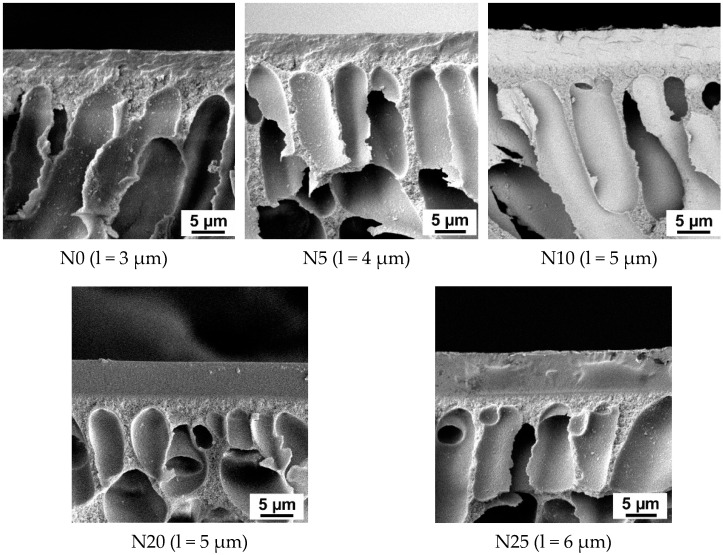
SEM microphotographs of TFC membrane cross-sections with different content of Al_2_O_3_·SiO_2_ nanoparticles.

The morphology of membranes was determined by applying scanning electron microscopy (SEM) (Figure 1). It was found that TFN membranes prepared by filtration of PVA aqueous solution with addition of Al_2_O_3_·SiO_2_ nanoparticles featured higher selective layer thickness compared to the reference TFC membranes. Moreover, membrane selective layer thickness increased with increasing Al_2_O_3_·SiO_2_ content in the PVA solution. For instance, when 5 wt.% of Al_2_O_3_·SiO_2_ nanoparticles are added, membrane selective layer thickness increased from 3 µm for the reference TFC membrane up to 4 µm for the N5 membrane (Figure 1). When 25 wt.% Al_2_O_3_·SiO_2_ nanoparticles were added, selective layer thickness increased twice (up to 6 µm) compared to the reference membrane (Figure 1). Since TFN membranes were obtained in the dynamic mode, the concentration polarization mechanism determines the selective layer formation. The concentration of retained substances (PVA macromolecules and aluminosilicate nanoparticles) significantly increases in a thin boundary layer of solution near the membrane surface due to concentration polarization. When the concentration of retained substances and nanoparticles reaches a certain value, the gel layer forms and precipitates on the membrane surface. This gel layer after cross-linking with GA and drying will form the membrane selective layer. Therefore, dead-end ultrafiltration mode for selective layer formation was selected to enhance the concentration polarization phenomenon and decrease the time of gel layer formation on the membrane surface. Correlation between kinetics of gel layer formation, preparation conditions, and separation performance for TFC PVA/PAN membranes was revealed in our previous study [21]. It is worth noting that the gel layer formed on the membrane surface due to concentration polarization acting as a secondary barrier to flow through the membrane. Addition of aluminosilicate nanoparticles to the PVA aqueous solution yields the hydrogen bond formation between silanol groups of nanoparticles and hydroxyl groups of PVA and donor–acceptor bond formation between free aluminum orbitals in aluminosilicate and lone pairs of electrons of oxygen atom in hydroxyl group in PVA. These bonds provide additional cross-linking of the gel layer. This cross-linked gel layer hinders transport through the membrane, enhancing concentration polarization and leading to more macromolecules and nanoparticles accumulating near the membrane surface. Moreover, aluminosilicate nanoparticles with PVA shell are larger compared to PVA macromolecules and predominantly do not pass through the membrane, accumulating in the gel layer. This increases the gel layer thickness. However, after some time of filtration a dynamic equilibrium between macromolecules and nanoparticles diffusing to the membrane surface and back to the bulk feed solution is established. Formation of hydrogen bonds and additional cross-linking retards the diffusion of PVA macromolecules back to the bulk solution and thicker gel layer is formed.

**Figure 2:**
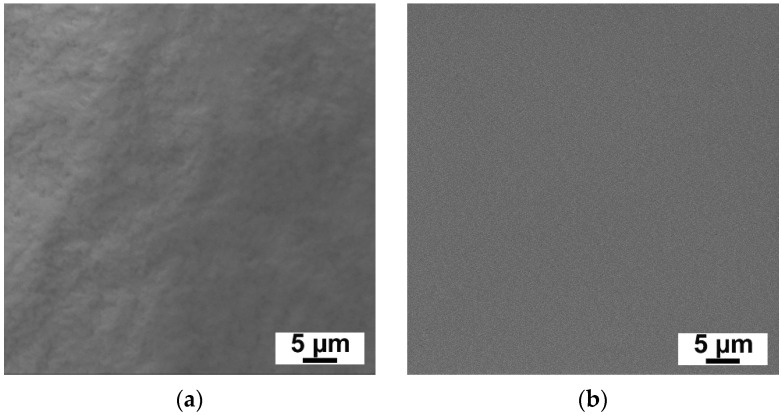
SEM microphotographs of selective layer surface of TFC membranes, content of Al_2_O_3_·SiO_2_ nanoparticles, wt.%: (**a**)—0, (**b**)—25 wt.%.

Selective layer surfaces of N0 and N25 membranes were investigated by SEM (Figure 2). It was found that no aggregates of Al_2_O_3_·SiO_2_ nanoparticles were observed on the membrane surface which confirms high dispersion degree of Al_2_O_3_·SiO_2_ in PVA aqueous solution and the small size of nanoparticles.

### 2.3. Atomic Force Microscopy

**Figure 3:**
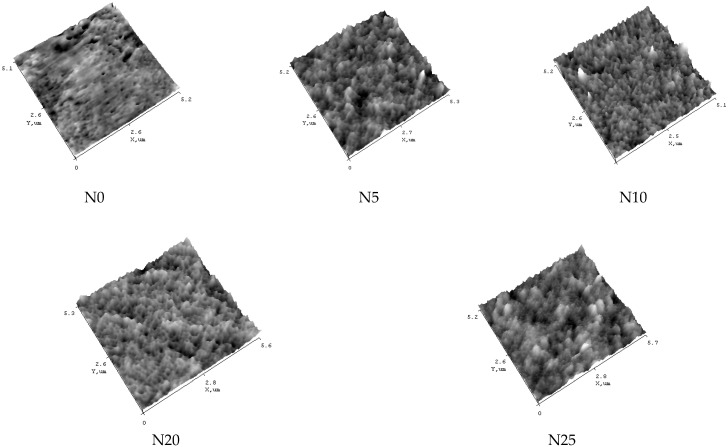
AFM images of selective layer surface of TFC PVA/PAN membrane (N0) and PVA-TFN Al_2_O_3_·SiO_2_/PAN membranes (N5, N10, N20, N25).

It was found that the N0 PVA/PAN membrane features smoother surface of the selective layer compared to the modified membranes (Figure 3, Table 2) which was confirmed by lower values of roughness parameters (R_a_ = 0.59 nm, R_q_ = 0.76 nm).

Overall, it was revealed that introduction of aluminosilicate nanoparticles into the selective layer results in the significant change in the topography of the membrane surface. It was shown that modification of the selective layer by Al_2_O_3_·SiO_2_ nanoparticles yields the formation of globules on the surface, which are nanoparticle conglomerates (Figure 3). These results are consistent with the increase in the average size of nanoparticles of aluminosilicate with the increase in concentration which was studied by dynamic light scattering technique and presented in Table 1. The size of globular formations increases with an increase in the concentration of Al_2_O_3_·SiO_2_ nanoparticles in the selective layer. This caused a nonmonotonic increase in the selective layer surface roughness (Table 2) with the maximum values of surface roughness for the N5 membrane. For the N25 membrane with the highest content of aluminosilicate nanoparticles, well-defined round globules cover the membrane surface. However, it is worth noting that surface roughness parameters are very low even for the N25 membrane with the highest concentration of aluminosilicate nanoparticles in the selective layer. It can be concluded that a dense layer is formed with minimum amount of nanoparticle conglomerates due to high degree of dispersion and uniform distribution of aluminosilicate nanoparticles in the PVA aqueous solution.

### 2.4. Contact Angle

It was found that the water contact angle of the selective layer surface tended to decrease with an increase in concentration of hydrophilic aluminosilicate nanoparticles (Table 3). It was pointed out that surface of the membrane selective layer became more hydrophilic. This could provide higher affinity of water to the membrane material. It is widely known that contact angle is influenced not only by the chemical nature of the surface but also by surface topography [62,63]. A relatively low decrease of water contact angle (from 78° to 69–74°) for N5, N10, N15 and N20 membranes is due to the increase in surface roughness of membrane surface (Table 2) which counterbalances the increase in the hydrophilicity with the increase in the content of aluminosilicate nanoparticles. When concentration of aluminosilicate nanoparticles reaches 25 wt.% of the PVA weight water contact angle significantly decreases down to 58°. It is due to the formation of large nanoparticle conglomerates which uniformly cover the membrane surface (Figure 3). It is worth noting that surface roughness is not very high for the N25 membrane, therefore an increase in the amount of hydrophilic formations on the membrane surface leads to the significant hydrophilization.

### 2.5. Membrane Performance

TFN PVA-Al_2_O_3_·SiO_2_/PAN membrane performance was investigated during ethanol dehydration via pervaporation as well as using water and dry ethanol as feed solutions. Membrane stability in pervaporation separation was studied by variation of water content (10, 20, 50, 80 wt.%) in feed solution and the temperature of feed solution (25, 35 and 50 °C).

It was expected that introduction of aluminosilicate nanoparticles into the PVA selective layer will increase water diffusivity and high adsorptive water selectivity over alcohols due to highly hydrophilic nature of aluminosilicate nanoparticles similarly to AS-SWNT reported in [56].

**Figure 4:**
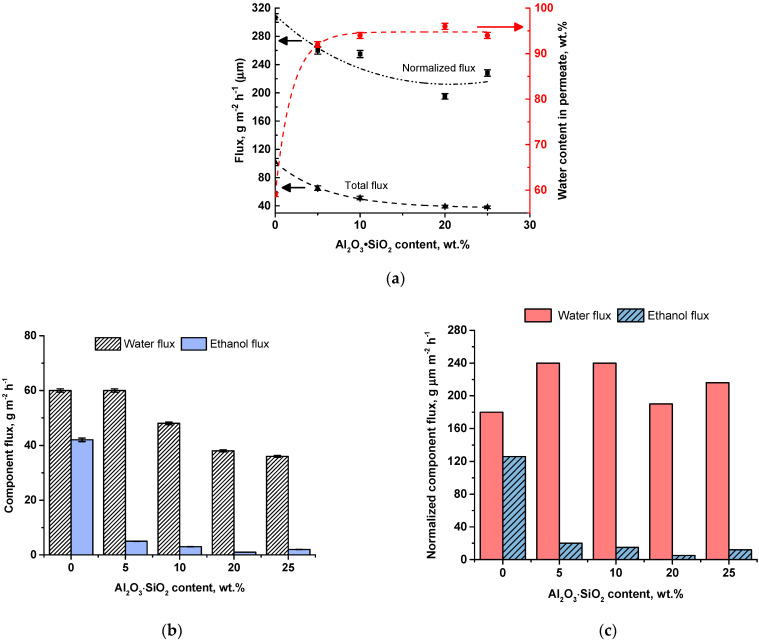
The dependence of TFC and TFN membrane total flux, thickness normalized flux and water content in permeate (**a**), component fluxes (**b**) and normalized component fluxes (**c**) on the content of Al_2_O_3_·SiO_2_ nanoparticles.

Flux of TFN PVA-Al_2_O_3_·SiO_2_/PAN membranes was found to become lower with increasing the aluminosilicate nanoparticle concentration in the membrane selective layer compared to the reference PVA/PAN TFC membrane (N0) (Figure 4a). Decreasing of membrane flux was a result of a thicker selective layer formation (Figure 1). It was shown that decline in water flux was significantly lower compared to the ethanol flux decline upon the increase in aluminosilicate nanoparticle concentration (Figure 4b). For instance, it was found that when Al_2_O_3_·SiO_2_ content reached 10 wt.%, selective layer thickness increases from 3 to 5 µm (by 40%) which leads to the total flux decline by 50% (from 102 down to 51 g·m^−2^·h^−1^) (Figure 1 and Figure 4a). However, water flux was found to decrease from 60 down to 48 g·m^−2^·h^−1^ (by 20%) and ethanol flux decreased from 42 to down to 3 g·m^−2^·h^−1^ (by 93%). It proves that addition of Al_2_O_3_·SiO_2_ nanoparticles limits ethanol flux through the membrane due to highly hydrophilic nature of aluminosilicate nanoparticles.

It was found that thickness normalized flux demonstrates similar dependence on the Al_2_O_3_·SiO_2_ content in the selective layer with the total flux (Figure 4a). However, the normalized flux of N20 TFN membrane was shown to be lower compared to the N25 membrane (Figure 4a). This is due to the higher selective layer thickness of N25 TFN membrane at almost equal flux values (Figure 1).

It was found that addition of aluminosilicate nanoparticles significantly increases water content in permeate due to significantly limiting ethanol transport. When 5 wt.% of aluminosilicate nanoparticles are added to PVA selective layer, water content in permeate increases from 59% to 92%. A further increase in Al_2_O_3_·SiO_2_ nanoparticle content in selective layer increases water content in permeate up to 94–96% with the maximum for the N20 membrane (Figure 4b).

Thickness normalized component fluxes were calculated and presented in Figure 4c. The normalized water fluxes for nanocomposite membranes were revealed to be higher compared to the reference membrane N0. It was found that the dependence of normalized ethanol flux on the Al_2_O_3_·SiO_2_ nanoparticle content in the selective layer was in accordance with the dependence of ethanol flux presented in Figure 4b,c. The normalized component fluxes for the N20 membrane were found to be slightly lower compared to other TFN membranes and the N25 membrane. Lower normalized component fluxes of the N20 membrane compared to N25 membrane were due to the combination of lower flux and lower selective layer thickness (5 µm) compared to the selective layer thickness of N25 (6 µm). The thicker selective layers of TFN membranes compared to the TFC N0 membrane resulted in the higher normalized fluxes (Figure 1 and Figure 4b,c).

**Figure 5:**
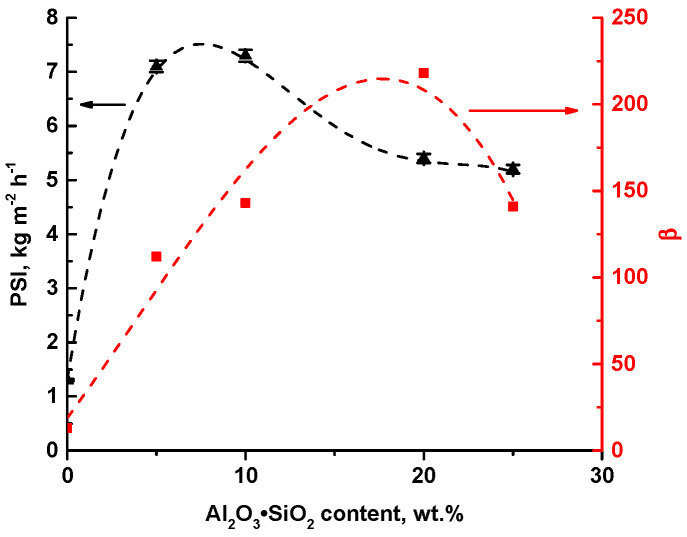
Dependence of PSI (black color) and separation factor (β) (red color) of PVA/PAN TFC membrane and PVA-Al_2_O_3_·SiO_2_/PAN TFN membranes on nanoparticle content in the selective layer.

It was shown that modification of the selective layer with Al_2_O_3_·SiO_2_ nanoparticles yielded an increase in separation factor (β) and pervaporation separation index (PSI) of TFN membranes in pervaporation separation of 90 wt.% ethanol/10 wt.% water mixture at 35 °C (Figure 5).

The dependence of separation factor (β) on the Al_2_O_3_·SiO_2_ content is shown in Figure 5. It was found that separation factor for TFN membranes was much higher (β = 112–218) compared to the reference N0 membrane (β = 13). Moreover, there was a maximum value for membrane N20. The maximum value was due to the fact that N20 is characterized by the highest water content in permeate.

N10 nanocomposite membrane demonstrated the highest PSI. It means that the N10 membrane is the most effective in the pervaporation of the selected mixture. However, PSI was revealed to decrease with addition of 20 wt.% Al_2_O_3_·SiO_2_ in relation to PVA weight. The PSI value for N20 was lower than for the N10 TFN membrane with a lower separation factor. This is due to the much lower flux of the N20 membrane compared to N10 membrane. It was found that TFN membranes modified with Al_2_O_3_·SiO_2_ nanoparticles demonstrated better separation performance in comparison with the reference PVA/PAN composite membrane.

**Figure 6:**
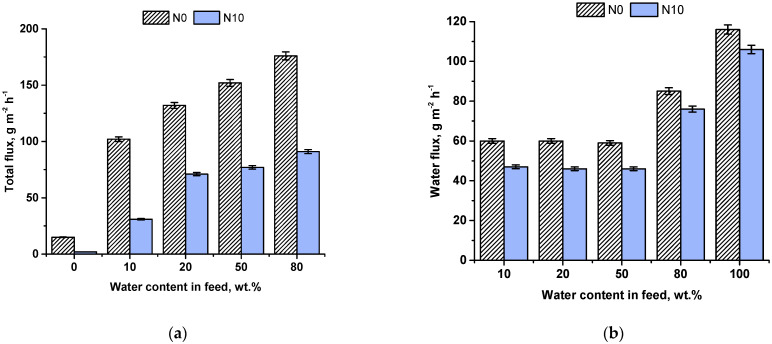

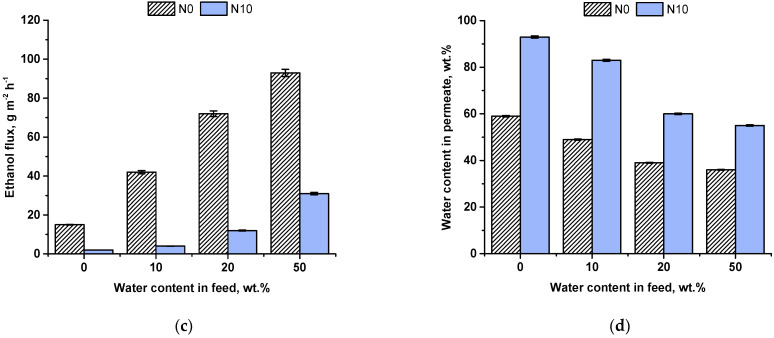
Dependence of total flux (**a**), water (**b**) and ethanol (**c**) fluxes, water content in permeate (**d**) of TFC and TFN membranes on the water content in the feed solution.

The N10 membrane was selected to study the stability in pervaporation compared to the reference TFC membrane due to the highest PSI value among other developed TFN PVA-Al_2_O_3_·SiO_2_/PAN membranes. Membrane stability was studied during ethanol dehydration with an increase in the water content in the feed solution (Figure 6). It was found that membrane flux increased with an increase in the water content in the feed solution for both the reference TFC N0 and modified TFN N10 membrane due the swelling in the feed mixture. Moreover, flux of TFN N10 membranes was shown to be two times lower compared to the reference N0 membrane (Figure 6a). Total flux was 102 and 51 g m^−2^ h^−1^ in dehydration of 90 wt.% ethanol feed solution and 176 and 91 g m^−2^ h^−1^ in dehydration of 20 wt.% ethanol feed solution for N0 and N10 membranes, correspondingly. It was revealed that water flux of the reference N0 membrane was slightly higher (by 20%) compared to the TFN N10 membrane (Figure 6b): 60 g m^−2^ h^−1^ for N0 membrane and 47 g m^−2^ h^−1^ for N10 membrane in pervaporation of 90 wt.% ethanol/10 wt.% water mixture. However, N0 TFC membrane demonstrates significantly higher ethanol flux, especially in pervaporation of feed mixtures with 10, 20 and 50 wt.% water (Figure 6c). Water content in permeate for N10 membrane was much higher compared to the reference N0 membrane in the whole range of investigated ethanol/water concentrations in the feed mixture (Figure 6d). As was mentioned previously, lower total flux of modified membranes N10 was a result of the increase in the selective layer thickness. Moreover, it is suggested that denser selective layer with higher degree of cross-linking is formed when Al_2_O_3_·SiO_2_ nanoparticles are introduced. A denser layer was formed due to the formation of donor–acceptor bonds between free aluminum orbitals in aluminosilicate and lone pairs of electrons of oxygen atom in hydroxyl group in PVA as well as hydrogen bonds between silanol groups of aluminosilicate and hydroxyl groups of PVA. Additional cross-linking with the introduction of aluminosilicate nanoparticles reduced membrane swelling in the feed mixture when water content increased. This also affects membrane selectivity. Cross-linking reduces segmental motion of macromolecules and free volume of the membrane selective layer which leads to obstruction of the molecule transfer and increasing the selectivity. Higher selectivity together with lower ethanol flux allowed concluding that the TFN N10 membrane features better stability to swelling compared to the reference membrane.

The pervaporation experiment with dry ethanol showed that TFC membrane N0 featured higher ethanol flux compared to TFN N10 membrane (Figure 6a,c). 

**Figure 7:**
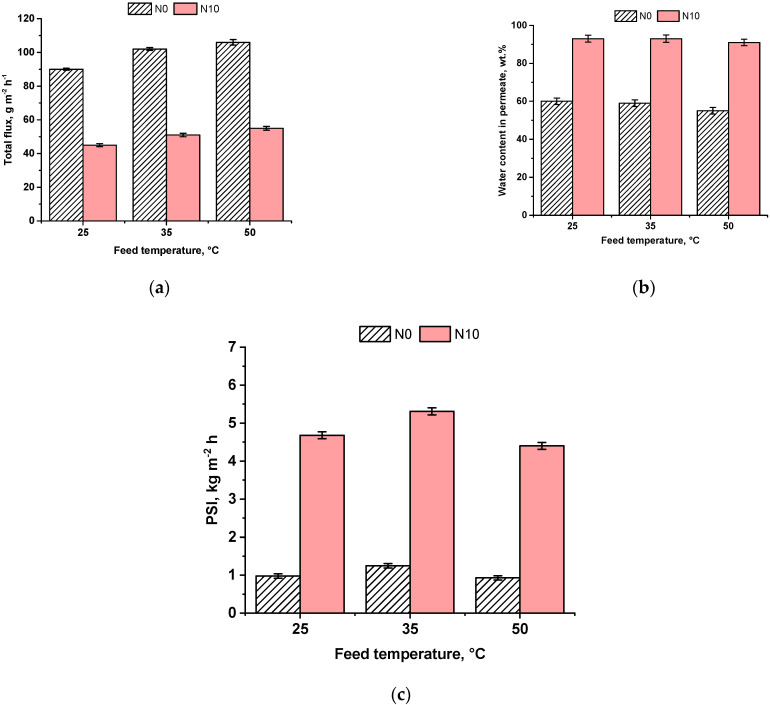
Total flux (**a**), water content in permeate (**b**) and PSI (**c**) of membranes versus the temperature of the feed solution in pervaporation of 90 wt.% ethanol/10 wt.% water mixture.

The effect of the temperature of TFC N0 and TFN N10 membranes was investigated in pervaporation of 90 wt.% ethanol/10 wt.% water mixture at 25, 35 and 50 °C (Figure 7).

It was found that membrane total flux increased with the increase in feed temperature due to the increase in vapor pressure (and chemical potential) which leads to the increase in driving force in pervaporation (Figure 7a). Moreover, the mobility of the diffusing molecules increases, which is attributed to the segmental motion of polymer chains. Water content in permeate (Figure 7b) was revealed to decrease from 60 to 55 wt.% for N0 membrane and from 93 to 91 wt.% for nanocomposite N10 membrane with the increase in the feed temperature from 25 °C to 50 °C. The total flux of the N10 membrane was shown to be two times lower compared to the reference N0 membrane over the whole studied range of temperatures. It was found that the developed N10 membrane is characterized by the much higher PSI compared to the reference N0 membrane (Figure 7c). Moreover, both N0 and N10 membranes are more effective in the dehydration of 90 wt.% ethanol/10 wt.% water mixture at 35 °C.

**Figure 8:**
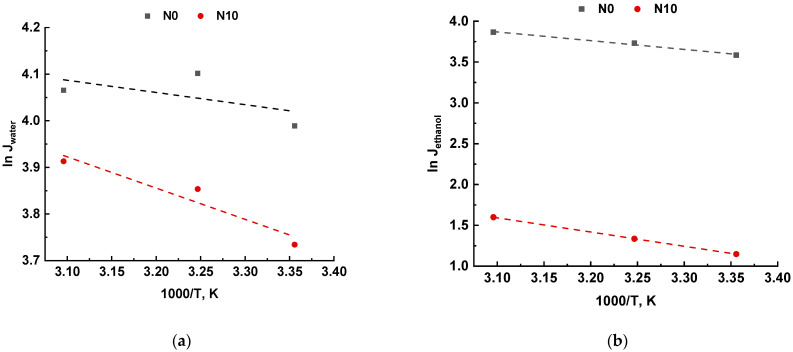
The dependence of natural logarithm of the partial water (**a**) and ethanol (**b**) fluxes on the temperature according to Arrhenius plot in pervaporation of 90 wt.% ethanol/10 wt.% water mixture.

The membrane performance could be also explained more in detail by taking into account the apparent activation energy. The apparent activation energy according to Equation (8) was counted from the slope of the Arrhenius plot (Figure 8).

The values of apparent activation energy for N0 and N10 membranes are presented in Table 4.

It was found that the apparent activation energy of water for both N0 and N10 membranes was lower compared to the apparent activation energy of ethanol, due to the smaller water molecules size and higher affinity of water to PVA. Therefore, water predominantly penetrates through the membrane selective layer. Moreover, higher apparent activation energy of ethanol demonstrates that ethanol flux is more dependent on temperature changes.

The apparent activation energy was revealed to become higher with addition of Al_2_O_3_·SiO_2_ nanoparticles into the membrane selective layer, due to the selective layer seals caused by the additional cross-linking PVA and Al_2_O_3_·SiO_2_ nanoparticles and increase of the selective layer thickness. As a result, the molecule transport slows down, and membrane flux declines (Figure 8a).

The comparison on properties of membranes prepared with introduction of Al_2_O_3_·SiO_2_ nanoparticles into the selective layer is presented in the Table 5.

The analysis showed that TFN membranes obtained in this work had comparable performance with membranes reported in the literature. However, in order to enhance TFN membrane flux, the selective layer thickness has to be reduced.

## 3. Materials and Methods

### 3.1. Materials

Polyacrylonitrile (PAN) ultrafiltration (UF) membranes based on the mixture of acrylonitrile homopolymer (M_n_ = 200 · 10^3^ g mol^−1^, Dolan, Germany) and a copolymer of acrylonitrile (AN) and methylacrylate (MA) (ratio of AN and MA units = 94:6, M_n_ = 80 · 10^3^ g mol^−1^, Dolan, Germany) reinforced with polyester nonwoven material were used as a membrane-support for TFC and TFN membrane preparation. UF PAN membrane pure water flux (PWF) and rejection of polyvinylpyrrolidone (PVP K-30, M_n_ = 40 · 10^3^ g mol^−1^, Fluka, Germany) are 400–500 L m^−2^ h^−1^ and 50–60% correspondingly.

Polyvinyl alcohol (PVA, M_n_ = 145 · 10^3^ g mol^−1^, 17–99, China) was used as a polymer for selective layer formation. PVA was cross-linked with glutaraldehyde (GA, 25 wt.%, Fluka, Germany) to prevent selective layer swelling, hydrochloric acid (HCl, 36 wt.% aqueous solution) served as a catalyst. Al_2_O_3_·SiO_2_ hydrosol (pH 2.8, concentration of dispersed phase 2.0 wt.%) was used as an additive in the selective layer of nanocomposite membranes.

### 3.2. Methods

#### 3.2.1. Synthesis of Al_2_O_3_·SiO_2_ Hydrosol

Synthesis of Al_2_O_3_·SiO_2_ hydrosol was conducted by neutralization of an aqueous solution of an aluminosilicate binding agent (ABA) on a strong acid cationite C100 (Purolite), previously prepared and converted to Na^+^ form [49,66,67]. An aqueous solution of an ABA with an assigned content Al_2_O_3_·SiO_2_ was preliminarily prepared in two stages: (i) synthesis of potassium aluminate (KAlO_2_) using potassium (KOH) and aluminum (Al(OH)_3_) hydroxides as precursors; (ii) synthesis of ABA using potassium aluminate (KAlO_2_) and sodium silicate (Na_2_SiO_3_) as precursors. All initial reagents (KOH, Al(OH)_3_, Na_2_SiO_3_) were produced by Sigma-Aldrich and had an analytical degree of purity, their solutions were prepared in ultrapure water (18.2 MΩ·cm). ABA solution was passed through a column with cationite (volume—300 mL, height—150 mm) with a linear velocity of 5 m h^−1^, and hydrosol Al_2_O_3_·SiO_2_ was obtained. The average particle size in aluminosilicate hydrosol was 39 nm, zeta-potential—−7.1 mV.

#### 3.2.2. Preparation of PVA Aqueous Solutions and PVA-Al_2_O_3_·SiO_2_ Dispersion

The 1.0 wt.% PVA aqueous solutions were obtained by dissolving of polymer at 80–90 °C for 3 h with a stirring rate of 300–400 rpm. The calculated amount of Al_2_O_3_·SiO_2_ hydrosol (nanoparticle content was calculated as the fraction of PVA weight in the solution) was added to the PVA aqueous solutions and mixed with a magnetic stirrer for 30 min. After that, the PVA/Al_2_O_3_·SiO_2_ solutions were sonicated for 60 min in an ultrasonic bath (Ultron, Poland, ν = 21 kHz). Further GA and HCl were added while stirring followed by the formation of the selective layer.

The dependence of average aluminosilicate nanoparticle size and zeta-potential in PVA aqueous solutions on the concentration of Al_2_O_3_·SiO_2_ were analyzed using Zetasizer ZS Nano (Malvern Panalytical, Malvern, UK).

#### 3.2.3. Preparation of PVA-Al_2_O_3_·SiO_2_/PAN TFN Membranes

The formation of a selective layer on an ultrafiltration PAN membrane-substrate was carried out by ultrafiltration of PVA solution and PVA-Al_2_O_3_·SiO_2_ aqueous dispersions with the addition of GA and HCl in dead-end mode at a transmembrane pressure of 0.3 MPa and temperature of 20 °C for 10 min. The Amicon-type ultrafiltration cell with an enlarged reservoir (400 mL) for feed solution was applied. The effective membrane area was 22.4 cm^2^. GA and HCl concentrations were 0.06 and 0.5 wt.% respectively. The conditions for membrane preparation were selected in accordance with the previous studies [20,21]. The initial ultrafiltration membrane-support was washed from glycerol upon ultrafiltration of distilled water (200–300 mL) at a pressure of 0.1 MPa and temperature of 20 °C before the selective layer formation.

Al_2_O_3_·SiO_2_ nanoparticle content in the solution was calculated as the fraction of the weight of PVA. After the selective layer formation, prepared TFN membranes were dried for 16 h at 50 °C in an oven. Membrane designations and preparation conditions are presented in Table 6.

#### 3.2.4. Scanning Electron Microscopy

The structure of the membrane cross-section was investigated by Phenom Pro scanning electron microscope (Thermo Fisher Scientific Inc., Waltham, MA, USA) at different magnifications. Membrane samples previously were cryogenically fractured in liquid nitrogen followed by the gold layer deposition.

#### 3.2.5. Atomic Force Microscopy

Topography of the membrane selective layer surface was investigated by an NT-206 atomic force microscope (AFM) (Microtestmachines, Gomel, Belarus) with standard silicon cantilevers NSC 11 A with the stiffness constant of 3 N m^−1^ (MikroMasch, Wetzlar, Germany).

The roughness Ra (nm) characterizes the variability along the Z surface at the scanning area of 5 × 5 μm and was estimated according to Equation (1):

(1) $$R_{a}=\frac{1}{N}\;\overset{N_{y}-1}{\underset{j=0}{\sum }}\overset{N_{x}-1}{\underset{i=0}{\sum }}\left|Z_{i,j}\;-\;\overset{-}{Z}\right|$$

where N—number of scan matrix points; Zi,j—height value in position (x, y); $\overset{-}{Z}$—arithmetic mean of the height at the whole of scanning area.

Root mean square deviation (R_q_, nm) was calculated by the Equation (2):

(2) $$R_{q}={\left(\frac{1}{N_{x}\cdot N_{y}}\;\overset{N_{y}-1}{\underset{j=0}{\sum }}\overset{N_{x}-1}{\underset{i=0}{\sum }}\left|Z_{i,j}\;-\;\overset{-}{Z}\right|\right)}^{1/2}$$

The roughness parameters were calculated by averaging values from 10–15 microphotographs from different places of the same membrane sample.

#### 3.2.6. Water Contact Angle Measurements

The hydrophilicity of membrane selective layer surface was studied by measuring water contact angle by sessile drop method using LK-1 goniometer (Otkrytaya nauka, Moscow, Russia). The measurements were repeated three times for each sample to minimize the experimental error.

#### 3.2.7. Pervaporation Experiments

Membrane transport properties were investigated in vacuum pervaporation of ethanol/water mixture with different component ratios in the feed solution: water content was 10, 20, 50, 80 wt.%, feed solution temperature was 35 °C. Moreover, pure water and ethanol were tested as feeds in pervaporation experiments. The dependence of membrane pervaporation performance on feed solution temperature was studied at 25 °C, 35 °C and 50 °C using 90 wt.% ethanol/10 wt.% water mixture as a feed. The compositions of the feed and permeate were analyzed using a gas chromatograph Crystal 5000 with a thermal conductivity detector (Chromatek, Yoshkar-Ola, Russia).

Total flux (J, g m^−2^ h^−1^) in pervaporation was calculated using Equation (3):

(3) $$J=\frac{\Delta m}{A\cdot \Delta t},$$

where Δm—permeate weight, g, A—effective membrane area, m^2^, Δt—experiment time, h.

Component (ethanol and water) fluxes (J_x_, g m^−2^ h^−1^) were determined according to the Equation (4):

(4) $$J_{x}=J\frac{C_{x}}{100},$$

where J—total flux, g m^−2^ h^−1^, Cx—ethanol/water content in permeate, wt.%.

Thickness normalized flux (J_N_, g µm m^−2^ h^−1^) was calculated using Equation (5) [68]:

(5) $$J_{N}=J\cdot L,$$

where J—total or component flux, g m^−2^ h^−1^, L—selective layer thickness, µm. Selective layer thickness was determined by SEM.

Separation factor β was determined according to Equation (6):

(6) $$\beta =\frac{Y_{i}/\left(1-Y_{i}\right)}{X_{i}/\left(1-X_{i}\right)},$$

where Xi and Yi—water content in feed solution and in permeate correspondingly.

Pervaporation separation index (PSI, kg m^−2^ h^−1^) indicating the effectiveness of the membrane in separation of the certain feed mixture was evaluated by Equation (7):

(7) PSI=J(β−1)/1000

The dependence of membrane flux on temperature is described by the Arrhenius relation (Equation (8)) [68]:

(8) $$J_{x}=J_{x,0}\exp\left(-\frac{E_{\mathrm{app}}}{\mathrm{RT}}\right),$$

where J_x_—the partial flux of components (ethanol and water) (g m^−2^ h^−1^), J_x,0_—the pre-exponential factor, E_app_—the apparent activation energy (J mol^−1^), R—the gas constant (J mol^−1^ K^−1^), T—the absolute temperature (K).

## 4. Conclusions

The novel PVA-Al_2_O_3_·SiO_2_/PAN TFN membranes were developed via ultrafiltration of aluminosilicate-PVA dispersion through the porous ultrafiltration membrane-support in dead-end mode at the transmembrane pressure of 0.3 MPa. The effect of aluminosilicate nanoparticle content in the selective layer on the structure and pervaporation performance was studied. It was found that introduction of the aluminosilicate nanoparticles into the PVA selective layer decreases membrane flux due to the increase in selective layer thickness. However, membrane selectivity toward water compared to ethanol significantly increased due to the presence of highly hydrophilic aluminosilicate nanoparticles in the selective layer structure. It was demonstrated that membrane modified with 10 wt.% of aluminosilicate nanoparticles demonstrates the highest separation pervaporation index in pervaporation dehydration of 90 wt.% ethanol-water mixture compared to other modified membranes. It was found that introduction of aluminosilicate nanoparticles yields higher swelling resistance of the membrane which is attributed to the additional cross-linking of the selective layer by hydrogen and donor-acceptor bonds.

## Figures and Tables

**Table 1:** Average size and zeta potential of Al_2_O_3_·SiO_2_ nanoparticles in 1 wt.% PVA aqueous solutions.

Al_2_O_3_·SiO_2_ Content, wt.% of PVA Weight	Particle Size, nm	Zeta Potential, mV
5	118	−3.92
10	142	−3.33
20	179	−3.31
25	186	−3.71

**Table 2:** The roughness parameters of selective layer surface of TFC PVA/PAN membrane and TFN PVA-Al_2_O_3_·SiO_2_/PAN membranes.

Membrane Designation	R_a_, nm	R_q_, nm
N0	0.59	0.76
N5	1.20	1.50
N10	0.80	0.93
N20	0.93	1.20
N25	0.80	0.97

**Table 3:** Water contact angle of membrane selective layer surface.

Membrane Designation	Water Contact Angle, °
N0	78
N5	74
N10	71
N20	69
N25	58

**Table 4:** The apparent activation energy of N0 and TFN N10 membranes.

Membrane Designation	E_app_ (Water), J mol^−1^	E_app_ (Ethanol), J mol^−1^
N0	2.18	8.91
N10	5.58	14.47

**Table 5:** The comparison of the transport properties of nanoparticle modified membranes for dehydration by pervaporation.

Membranes	Selective Layer Thickness [µm]	Mixture	Water Content in Feed [wt.%]	Temperature [°C]	Permeation Flux [g m^−2^ h^−1^]	Water Content in Permeate [wt.%]	Reference
PVA-20 wt.%SiO_2_/PAN support	6	Ethanol/water	10	34	44	89	[20]
PVA-2.5 wt.% sodium aluminasilicate	25	1,4-dioxane-water	10	30	150	85	[57]
PVA-15 wt.%SiO_2_/PAN support	25	Ethanol/water	10	60	1193	78.5	[64]
Sodium alginate—15 wt.% magnesium aluminum silicate particles	-	Isopropanol/water	10	30	56	99.95	[55]
PVA-3 wt.% Fe(II)/Fe(III)/polyester fabrics	-	Isopropanol/water	10	30	82	94	[65]
PVA-Al_2_O_3_·SiO_2_/PAN	5	Ethanol/water	10	35	51	94	This work

**Table 6:** Membrane designations and preparation conditions.

Membrane Designation	Al_2_O_3_·SiO_2_ Content, wt.% of PVA Weight
N0	0
N5	5
N10	10
N20	20
N25	25

## Data Availability

The data presented in this study are available on request from the corresponding author. The data are not publicly available due to being a part of ongoing research.
